# Body Mass Index and Waist Circumference Cut-Points in Multi-Ethnic Populations from the UK and India: The ADDITION-Leicester, Jaipur Heart Watch and New Delhi Cross-Sectional Studies

**DOI:** 10.1371/journal.pone.0090813

**Published:** 2014-03-05

**Authors:** Danielle H. Bodicoat, Laura J. Gray, Joseph Henson, David Webb, Arvind Guru, Anoop Misra, Rajeev Gupta, Naval Vikram, Naveed Sattar, Melanie J. Davies, Kamlesh Khunti

**Affiliations:** 1 University of Leicester, Diabetes Research Centre, Leicester, Leicestershire, United Kingdom; 2 University of Leicester, Department of Health Sciences, Leicester, Leicestershire, United Kingdom; 3 Postgraduate Institute of Medical Education and Research, Chandigarh, Punjab, India; 4 Fortis Hospitals, Department of Diabetes and Metabolic Diseases, New Delhi, Delhi, India; 5 Fortis Escorts Hospital, Department of Internal Medicine, Jaipur, Rajasthan, India; 6 All India Institute of Medical Sciences, Department of Medicine, New Delhi, Delhi, India; 7 University of Glasgow, Institute of Cardiovascular and Medical Sciences, Glasgow, United Kingdom; Uppsala Clinical Research Center, Sweden

## Abstract

**Aims:**

To derive cut-points for body mass index (BMI) and waist circumference (WC) for minority ethnic groups that are risk equivalent based on endogenous glucose levels to cut-points for white Europeans (BMI 30 kg/m^2^; WC men 102 cm; WC women 88 cm).

**Materials and Methods:**

Cross-sectional data from participants aged 40–75 years: 4,672 white and 1,348 migrant South Asian participants from ADDITION-Leicester (UK) and 985 indigenous South Asians from Jaipur Heart Watch/New Delhi studies (India). Cut-points were derived using fractional polynomial models with fasting and 2-hour glucose as outcomes, and ethnicity, objectively-measured BMI/WC, their interaction and age as covariates.

**Results:**

Based on fasting glucose, obesity cut-points were 25 kg/m^2^ (95% Confidence Interval: 24, 26) for migrant South Asian, and 18 kg/m^2^ (16, 20) for indigenous South Asian populations. For men, WC cut-points were 90 cm (85, 95) for migrant South Asian, and 87 cm (82, 91) for indigenous South Asian populations. For women, WC cut-points were 77 cm (71, 82) for migrant South Asian, and 54 cm (20, 63) for indigenous South Asian populations. Cut-points based on 2-hour glucose were lower than these.

**Conclusions:**

These findings strengthen evidence that health interventions are required at a lower BMI and WC for South Asian individuals. Based on our data and the existing literature, we suggest an obesity threshold of 25 kg/m^2^ for South Asian individuals, and a very high WC threshold of 90 cm for South Asian men and 77 cm for South Asian women. Further work is required to determine whether lower cut-points are required for indigenous, than migrant, South Asians.

## Introduction

There is an extensive literature showing that high levels of adiposity are related to morbidity and mortality. This has resulted in leading health organisations recommending weight loss interventions for obese individuals, who are typically identified using body mass index (BMI) and/or waist circumference (WC) as both measures are strongly correlated with body fat and are simple to measure [1], [2]. Moreover, there are recommendations for certain procedures, such as bariatric surgery [2], and treatments, such as GLP-1 [3], that are based on BMI cut-points for severe obesity.

BMI is calculated as weight in kilograms divided by height in metres squared, and is often categorised for ease of interpretation. The World Health Organisation (WHO) defines the following cut-points for BMI: <18.5 kg/m^2^ underweight, 18.5–24.9 kg/m^2^ healthy weight, 25.0–29.9 kg/m^2^ overweight, and ≥30 kg/m^2^ obese [1]. These cut-points were based on visual inspection of the relationship between BMI and mortality, which tends to be J or U shaped, and guidelines suggest weight loss interventions when BMI reaches at least 25 kg/m^2^, with a greater focus on 30 kg/m^2^ or higher [1]. Despite its wide spread use, it is acknowledged that BMI has limited use in some populations, such as very muscular individuals [2]. Moreover, BMI tends to reflect overall adiposity whereas research suggests that abdominal adiposity may independently influence health outcomes [4], [5]. Consequently, the use of recommendations based on measurements that reflect abdominal adiposity, such as WC, is increasing. Recommended cut-points to define a very high WC are 102 cm for men and 88 cm for women [2], [6]. These cut-points were derived based on their ability to detect an obese BMI, rather than on their relationship with health outcomes [7]. Though both BMI and WC have their limitations, both measures independently contribute to the prediction of non-abdominal, abdominal subcutaneous and visceral fat.

The derivation of the BMI and WC cut-points mostly used data from Western European or American populations [1]. There is growing interest in the validity of these cut-points in other populations. In particular, it has been debated whether these cut-points can be applied to other ethnic groups in whom the distribution of body fat tends to be different to white populations [8], [9]. Indeed, studies have shown that ethnic minority populations have a similar level of health risk at lower adiposity thresholds than white populations [10]. It is vital that appropriate cut-points are defined for ethnic minority populations because they develop more obesity-related problems at a younger age, and lead to worse outcomes, therefore earlier intervention may be necessary in this population [11]. Work has already been done in this area and other studies have estimated that equivalent BMI cut-points for South Asian groups range between 21 and 29 kg/m^2^ [12]–[14], with expert groups recommending cut-points of 25 kg/m^2^ and 27.5 kg/m^2^ [15]–[17]. At present, no expert groups recommend different WC thresholds for black and minority ethnicity groups. It is important to also assess BMI and WC cut-points in both migrant and indigenous populations, something which has not been done previously, since it has been shown that migrant South Asians (MSAs) have a higher prevalence of dysglycaemia and cardiovascular disease risk factors compared with indigenous South Asians (ISAs) [18], [19].

We therefore used international data from the UK and India to derive BMI and WC cut-points for South Asian groups that are risk equivalent to the current thresholds in terms of glucose levels for white groups.

## Materials and Methods

### Study Population

These analyses used data from three population based studies; one from the UK (ADDITION-Leicester) and two from India (Jaipur Heart Watch (JHW-2) and New Delhi studies). The findings from ADDITION-Leicester are an update of a previous analysis [20]. Ethical approval was obtained from the University Hospitals of Leicester (UHL09320) and Leicestershire Primary Care Research Alliance (64/2004) local research ethics committees for ADDITION-Leicester, from the Institutional Ethics Board of the All India Institute of Medical Sciences for the New Delhi study, and from the Institutional Ethics Committee of Monilek Hospital and Research Centre for JHW-2. All participants provided written informed consent.

The ADDITION-Leicester study is a UK-based two phase study that has been described in detail elsewhere (2005–2009) [21]. The first phase was a population level study where people were screened for type 2 diabetes mellitus, followed by the second phase where screened individuals with type 2 diabetes mellitus were enrolled into a randomised controlled trial. Only data from the first stage were used in these analyses. All general practices in the Leicestershire and Rutland Strategic Health Authority, an area with a large South Asian population [22], were invited to participate and those that agreed were asked to identify patients that met the inclusion and exclusion criteria for the study. The inclusion criteria were that participants must be aged 40–75 years inclusive if they were of White European ethnicity and 25–75 years inclusive if they were of Asian, Black or Chinese ethnicity. Exclusion criteria included previous diagnosis of diabetes, being housebound, presence of a terminal illness, active psychotic illness, pregnancy or lactation. A random sample of eligible individuals was then sent an invitation pack and a pre-screening questionnaire. Invitation packs were available in English, Hindi, Gujarati, Urdu and Punjabi. Those responding to this letter were invited to a screening appointment [21]. Participants classified their ethnicity into one of the categories in the 2001 national census. People who identified themselves as being in an ethnic group other than White or South Asian were not included in these analyses due to the small number of study participants in these groups. Of the participants screened in the ADDITION-Leicester study (n = 6749; response rate = 22%), we excluded from these analyses those who were younger than 40 years of age (n = 359), those whose ethnic group was unknown (n = 203) or was not White (i.e. white British, white Irish, or any other white background) or South Asian (i.e. of Indian, Pakistani, Bangladeshi, or Sri Lankan ethnicity; n = 146), those whose WC and BMI were missing (n = 8) and those with no fasting or 2-hour glucose data (n = 20). Thus, 6013 ADDITION-Leicester participants were included in these analyses.

The JHW-2 Study recruited adults aged 20–75 years (n = 1123; response rate = 62%) for a population based epidemiological study that has been described elsewhere [23]. The JHW Study was conducted in 2001 in Jaipur, Rajasthan, Northern India and involved house to house surveys. Only participants aged 40–75 were included in these analyses (n = 605) and those with both WC and BMI missing were excluded (n = 1) as were those with missing fasting glucose (n = 13) meaning that 591 JHW-2 participants were included.

Subjects for the New Delhi cohort (n = 1044; response rate = 65%) were randomly selected from various residential colonies as described previously (1998–2003) [24]. Care was taken to have approximate representation from each income group (high income group ∼10%, middle income group ∼65–70%, and low income group ∼15–20%). As an initial step, a list of the number of houses along with the number of adult subjects in each household was obtained from each locality. Subsequently, a household was selected for participation in the study using a random number list that was generated. Only one individual from one household was selected. Subjects with diabetes, any severe acute or chronic illness, known human immunodeficiency virus (HIV) seropositivity and pregnant and lactating women were excluded from the study. Only participants aged 40–75 were included in these analyses (n = 402) and those with missing fasting glucose (n = 8) meaning that 394 New Delhi participants were included. WC and BMI were recorded and available for all New Delhi participants. All JHW-2 and New Delhi participants were assumed to be of South Asian ethnicity.

### Variables

In all three studies, fasting venous blood samples were obtained after a minimum fast of eight hours, and samples were analysed at single laboratories within the three study settings. Glucose was measured using the glucose peroxidase method in JHW-2 [25], the hexokinase enzymatic method (Abbott Aeroset clinical chemistry analyser) in ADDITION-Leicester [21], and colorimetrically using GOD/PAP test kit (Randox Laboratory, San Francisco, CA, USA) in the New Delhi cohort. In ADDITION-Leicester, data were also available on 2-hour glucose after consumption of a standard 75 g dose of glucose in an oral glucose tolerance test. Oral glucose tolerance tests were not administered in the two Indian studies, thus 2-hour glucose is not available in those studies.

In all three studies, anthropometric measurements were objectively recorded, BMI (kg/m^2^) was calculated as weight in kilograms divided by height in metres squared, and WC was measured at the mid-point between the lower costal margin and the level of the anterior superior iliac crest.

### Statistical Analysis

The aim of these analyses was to ascertain BMI and WC cut-points for South Asian groups that are risk equivalent in terms of glucose levels to the currently used standards that were derived in white populations. For BMI, this cut-point is 30 kg/m^2^ for obesity [1], [2] and for a raised WC these cut-points are 102 cm for men and 88 cm for women [2], [6]. Since BMI cut-points are not currently gender-specific, analyses regarding BMI were performed on the population as a whole. Conversely, WC cut-points as currently defined are gender-specific and so the fractional polynomial models described below were fitted separately for men and women for WC. All analyses were performed in Stata v12.1 and an alpha level of 5% was treated as statistical significance.

Fractional polynomial models were fitted with fasting and 2-hour glucose (continuous) as the outcome in turn, and ethnicity (categorical: white, MSA, ISA), adiposity (BMI or WC; continuous) and an interaction between adiposity and ethnicity (continuous) as the explanatory variables. Additionally each model was adjusted for age only. The fractional polynomial model tests linear and non-linear terms for the continuous variables and selects the best fitting, most parsimonious terms for the final model. The interaction term was included as it allows the relationship between the anthropometric variables and glucose to differ by ethnic group. During model testing, it was found that fasting and 2-hour glucose were non-Normally distributed and so they were transformed by adding 10 and then taking the natural logarithm; these transformed variables were used as the outcome to improve the model fit.

File S1 provides a detailed explanation of the methods used to derive the cut-points and their confidence intervals. Briefly, the fitted values were used to find the mean glucose value (G) for a white individual with a BMI of 30 kg/m^2^ or WC of 88 cm or 102 cm for women and men, respectively. The equivalent BMI/WC cut-points in the South Asian groups were then found by identifying the BMI/WC in those groups for which the mean glucose value was equal to G. As in a previous study [13], a 95% confidence interval (CI) was estimated using a method similar to the fiducial approach. This involved finding the point on the lower and upper confidence bands where the mean glucose value was G and using the corresponding BMIs/WCs as the upper and lower estimates of the CI, respectively.

## Results

There were a total of 6998 participants in these analyses (4667 White, 1346 MSAs, 985 ISAs) and their characteristics are summarised in Table 1. There were significant differences between the ethnic groups for all the baseline characteristics considered. Notably, for both men and women, MSAs had lower or similar BMI and WC than white individuals, and ISAs had even lower values than MSAs.

**Table 1:** Summary characteristics of the study population by ethnicity and sex.

	Men				Women			
	White	Migrant South Asians	Indigenous South Asians		White	Migrant South Asians	Indigenous South Asians	
Characteristic	Mean (SD)	Mean (SD)	Mean (SD)	P value^a^	Mean (SD)	Mean (SD)	Mean (SD)	P value^a^
Age, years	58.7 (9.5)	53.9 (9.1)	51.4 (9.0)	<0.001	58.5 (9.5)	52.2 (8.2)	51.8 (9.4)	<0.001
Body mass index, kg/m^2^	28.2 (4.2)	26.6 (4.1)	23.8 (5.2)	<0.001	28.4 (5.6)	28.5 (5.3)	24.1 (6.2)	<0.001
Waist circumference, cm	100.0 (11.4)	95.8 (10.2)	92.1 (15.3)	<0.001	89.9 (13.4)	89.6 (12.2)	84.0 (14.4)	<0.001
	**Median (IQR)**	**Median (IQR)**	**Median (IQR)**	**P value** b	**Median (IQR)**	**Median (IQR)**	**Median (IQR)**	**P value** b
Fasting blood glucose, mmol/L	5.2 (4.9, 5.5)	5.2 (4.9, 5.6)	5.0 (4.4, 5.8)	<0.001	4.9 (4.7, 5.3)	5.0 (4.7, 5.4)	5.0 (4.5, 5.7)	0.037
2-hour glucose, mmol/L	5.3 (4.3, 6.7)	5.9 (4.7, 7.3)		<0.001	5.6 (4.6, 6.7)	5.9 (4.9, 7.2)		<0.001
	**N (%)**	**N (%)**	**N (%)**	**P value** c	**N (%)**	**N (%)**	**N (%)**	**P value** c
Current smoker								
Yes	387 (17.6)	104 (15.9)	124 (36.7)		359 (14.7)	3 (0.4)	123 (22.8)	
No	1796 (82.4)	551 (84.1)	214 (63.3)	<0.001	2092 (85.4)	682 (99.6)	417 (77.2)	<0.001
**Total**	**2199 (100.0)**	**660 (100.0)**	**387 (100.0)**		**2468 (100.0)**	**686 (100.0)**	**598 (100.0)**	

Abbreviations: IQR, Interquartile range; SD, Standard deviation.

Number of missing values: 0 Age, 24 Body Mass Index, 60 Waist circumference, 9 Fasting glucose,29 2-hour glucose in Addition-Leicester and 985 in JHW as 2-hour glucose was not measured in that study, 0 Sex, 149 Current smoker.

a P-values test the difference between the three ethnic groups and were derived using analysis of variance.

b P-values test the difference between the three ethnic groups and were derived using Kruskal-Wallis tests.

c P-values test the difference between the three ethnic groups and were derived using chi-squared tests.

Table S1 and the Figures S1, S2, S3, S4, S5 and S6 in File S2 show the fitted models which were used to derive cut-points, and the derived cut-points and their confidence intervals are shown in Table 2. In both South Asian groups the cut-points equivalent to 30 kg/m^2^ in the white group were lower at 25 kg/m^2^ based on fasting glucose and 21 kg/m^2^ based on 2-hour glucose for MSAs, and 18 kg/m^2^ for ISAs. Likewise, among men, the South Asian groups had a lower cut-point for WC (102 cm in white men) at 79 cm and 90 cm in MSAs and 87 cm in ISAs. Similarly, SA women had a lower cut-point for WC (88 cm in white women) at 72 cm and 77 cm in MSAs and 54 cm in ISAs.

**Table 2:** Body mass index and waist circumference cut-points for people of South Asian ethnicity equivalent in terms of glucose levels to those in people of white ethnicity.

	Age-adjusted cut-point (95% Confidence Interval)	
Measurement	Migrant South Asians	Indigenous South Asians
**Obese BMI (kg/m^2^) in men and women** a		
Fasting glucose	24.9 (23.5, 26.4)	18.2 (16.3, 19.7)
2-hour glucose^b^	21.1 (18.7, 22.7)	
**Waist (cm) in men** a		
Fasting glucose	90.0 (85.0, 94.6)	87.1 (82.3, 91.2)
2-hour glucose^b^	78.8 (67.8, 84.5)	
**Waist (cm) in women** a		
Fasting glucose	77.1 (70.7, 82.4)	53.5 (12.7, 63.4)
2-hour glucose^b^	71.7 (63.8, 76.6)	

Abbreviations: BMI, Body mass index.

a White European equivalents: BMI = 30 kg/m^2^, male waist circumference = 102 cm, female waist circumference = 88 cm.

b 2 hour glucose was not measured in the indigenous South Asian study population.

## Discussion

This study found that South Asian individuals have higher glucose levels than white individuals at equivalent levels of BMI and WC, adding further support that health interventions should occur at lower adiposity levels in these groups.

The limitations of our work should be considered when interpreting the results. The primary limitation is that these analyses were based on cross-sectional studies and so we were unable to estimate cut-points based on future health outcomes, such as cardiovascular disease or mortality. Instead, we based the cut-points on fasting and 2-hour glucose. These outcomes were chosen as markers of future health outcomes because people with high glucose levels are known to have a high risk of progressing to overt diabetes [26], as well as a high risk of developing the micro- and macro-vascular complications associated with diabetes [27]. Moreover, fasting glucose and 2-hour glucose appear to reflect different underlying biomedical mechanisms, with 2-hour glucose largely influenced by peripheral insulin resistance, and both measures detect hyperglycaemia in different groups of individuals to some extent [28]. Thus, it is important to use both, rather than a single measure. This approach of using diabetes and cardiovascular risk factors, such as high glucose, to derive cut-points is a common one that has been used in several studies previously [14].

While a major strength of our study was the ability to combine international data from studies conducted in India and the UK, inevitably differences in methodology were present, which need to be considered when interpreting our results. The primary difference is the methods used to assay glucose. To understand the potential effect of this difference we performed sensitivity analyses to determine how different the Indian methods would need to be from the UK ones before our conclusions changed (data not shown). In these analyses we found that the Indian methods could be up to −0.5 mmol/l different on average from the ADDITION-Leicester method and our conclusion that ISAs require a lower BMI cut-point than White Europeans would still stand. For context, in the UK, these methods differed by an average of −0.3 mmol/l in unpublished quality control data from NEQAS. Therefore, our results appear to be robust to the differences in assaying glucose. Furthermore, the direction of the cut-points for MSAs and ISAs compared with White Europeans is the same which provides further reassurance regarding the validity of combining the datasets. However, due to these differences, the estimates for MSAs might be less precise than those for ISAs.

Aside from the international data, the strengths of our study include the large overall sample size, the investigation of non-linear associations, and the accurate, objective measurement of anthropometric and glycaemic variables. The findings from ADDITION-Leicester are an update of a previous analysis [20], with the novel aspects of these results being the investigation of non-linear relationships between adiposity and glucose, and separate consideration of fasting and 2-hour glucose. Furthermore, the inclusion of the ISA population is highly novel with no existing research comparing cut-points in migrant and indigenous South Asians.

In South Asians, we found lower cut-points for obesity in both the migrant (25 kg/m^2^) and indigenous (18 kg/m^2^) populations based on fasting glucose. The cut-point for MSAs based on 2-hour glucose was lower at 21 kg/m^2^. Importantly, while some of the errors of margin were fairly wide, none of them included the cut-point for the white population further suggesting that the BMI cut-points for South Asians should be lowered. Careful consideration around recommended cut-points is needed from a public health point of view as cut-points that are too low could potentially place an unmanageable burden on health care providers and could be demotivating for individuals who are unable to attain a BMI below the obesity threshold. Thus, there is an argument for recommending our highest threshold of 25 kg/m^2^ for obesity in South Asians. This is consistent with other estimates which tend to range between 21 and 29 kg/m^2^, with most estimates between 23 and 27 kg/m^2^ [12]–[14]. Moreover, it is consistent with, albeit at the lower end of the range, the cut-points of 25 kg/m^2^ and 27.5 kg/m^2^ that have been recommended by expert groups [15]–[17]. It could be that different cut-points are appropriate for MSAs and ISAs but, since this is the first study to compare cut-points for ISAs and MSAs, further work is required before different cut-points could be recommended, and even then it might not be preferable to have separate cut-points if those for ISAs are detrimentally low. It is notable that ISAs had lower BMI and WC than MSAs, which is consistent with previous studies that have shown a higher prevalence of cardiovascular disease risk factors in MSAs than ISAs [18], [19]. This difference could also reflect the rise in obesity over time since the data for MSAs were collected later (2005–2009) than that for ISAs (1998–2003). Among South Asians, in both men and women, the WC cut-point was consistently lower in South Asians than in whites with none of the CIs including the existing WC cut-points of 102 cm or 88 cm. Therefore, our results for WC in South Asians are indicative that WC thresholds should be lowered for this population. At present, no expert groups recommend different WC thresholds for black and minority ethnicity groups, although the UK-based National Institute for Health and Care Excellence (NICE) have recently considered this issue [29]. Based on our findings, we would recommend a WC threshold of 90 cm for men and 77 cm for women. Whether the lower BMI and WC cut-points for South Asians are due to an increased genetic predisposition or changes in lifestyle remains unclear. Information derived from large longitudinal studies comparing migration-induced differences in risk factor exposures and disease outcomes will be helpful in understanding the temporal link between precise environment mechanisms and genetic interactions that are known to accelerate morbidity and mortality attributable to metabolic risk.

In conclusion, this study derived cut-points for BMI and WC in South Asian populations living in the UK and in India that were lower than those derived in white populations. Our findings add to existing evidence that cut-points should be lower for South Asian than white populations. Further work is required to determine whether lower cut-points are required for indigenous, than migrant, South Asians.

**Data availability:** None.

## Supporting Information

File S1 **Method for finding cut-points and their confidence intervals.**
(DOCX)

File S2 **Table S1 and Figures S1–S6.**
(DOCX)
