# Supplementary material for: Body Mass Index and Waist Circumference Cut-Points in Multi-Ethnic Populations from the UK and India: The ADDITION-Leicester, Jaipur Heart Watch and New Delhi Cross-Sectional Studies
Source: PLoS One. 2014 Mar 5;9(3):e90813. doi: 10.1371/journal.pone.0090813 (PMC3944886; doi:10.1371/journal.pone.0090813)
Supplement: File S1 — Method for finding cut-points and their confidence intervals. (DOCX) [file pone.0090813.s001.docx]

**File S1. Method for finding cut-points and their confidence intervals.**

The method for finding the BMI cut-point for the migrant South Asian group from Figure 1 is as follows. Transformed fasting glucose for a BMI of 30kg/m^2^ in the white group can be read off the graph at the intersection of Line and the fitted line for the white group as 2.718 (Line A). The equivalent BMI cut-point for the migrant South Asian group can be read from where Line A crosses the fitted line for the migrant South Asian group as 24.9kg/m^2^ (Line B). Identical methodology can be used to find the cut-points for the indigenous South Asian group.

**
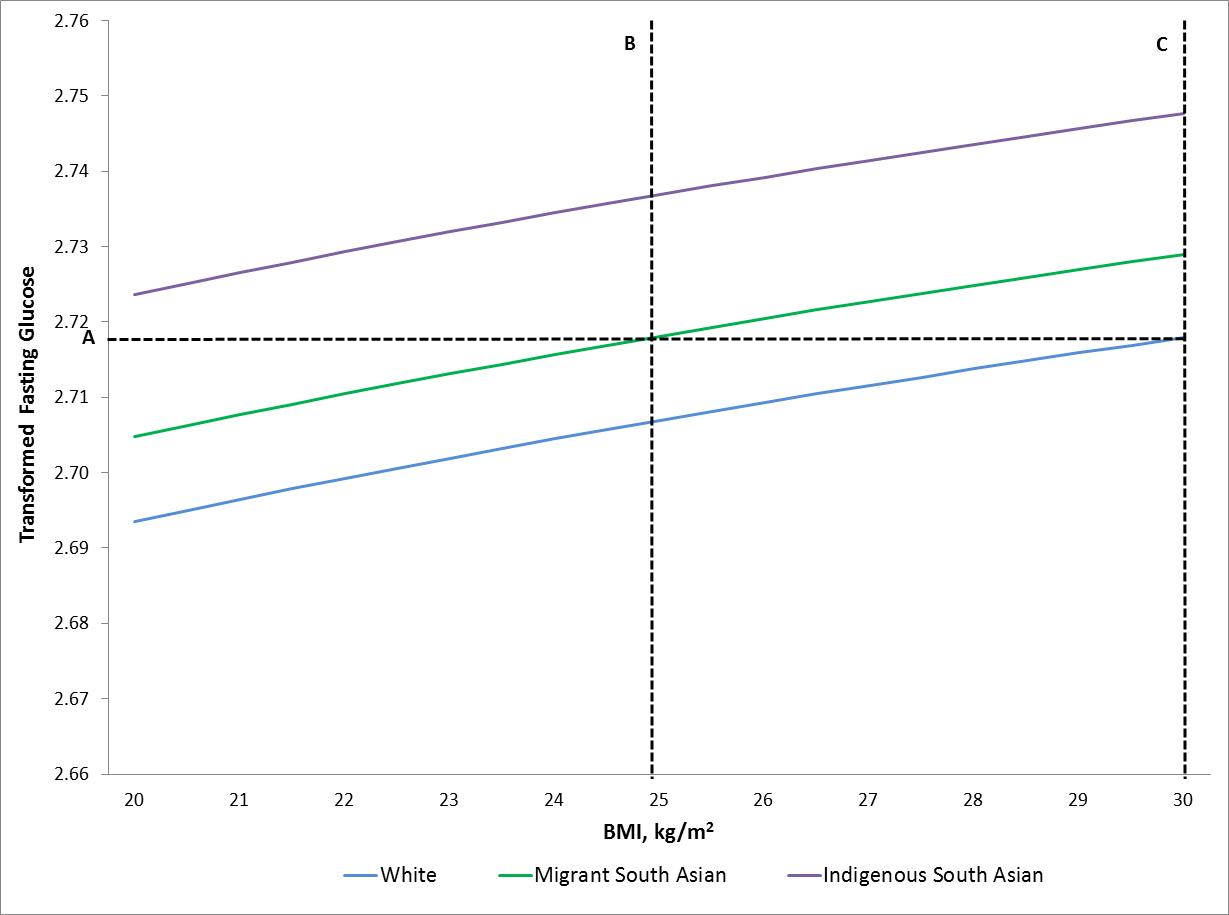
**

**Figure 1.** The age-adjusted relationship between body mass index (BMI) and fasting glucose by ethnic group.

Similar methods were used to find the confidence intervals; an approach similar to the fiducial approach. As before, Figure 2 shows line A at the average transformed fasting glucose value for a BMI of 30 kg/m^2^ in the white group, the green line shows the relationship between BMI and fasting glucose in the migrant South Asian group, and line B shows the cut-point estimate for BMI in the migrant South Asian group. Additionally, Figure 2 also shows the confidence bands for the association in the migrant South Asian group (for simplicity the confidence bands for the other ethnic groups are not shown). The upper confidence limit is found by identifying the point where line A intersects with the lower confidence band for the migrant South Asians. Likewise, the lower confidence limit is found by identifying the point where line A intersects with the upper confidence band for the migrant South Asians. The resulting CIs are not symmetrical because the way in which they are derived is more similar to a Bayesian approach, i.e. they are derived internally from the observed data, as opposed to a standard Frequentist approach which uses equations that impose symmetry to estimate CIs.

In practice, the cut-points and their confidence intervals were found by solving the equations in Supplementary Table 1, which fully describe the lines in the graphs, as this gives more precise values than reading from the graph, but the principles are the same.

**
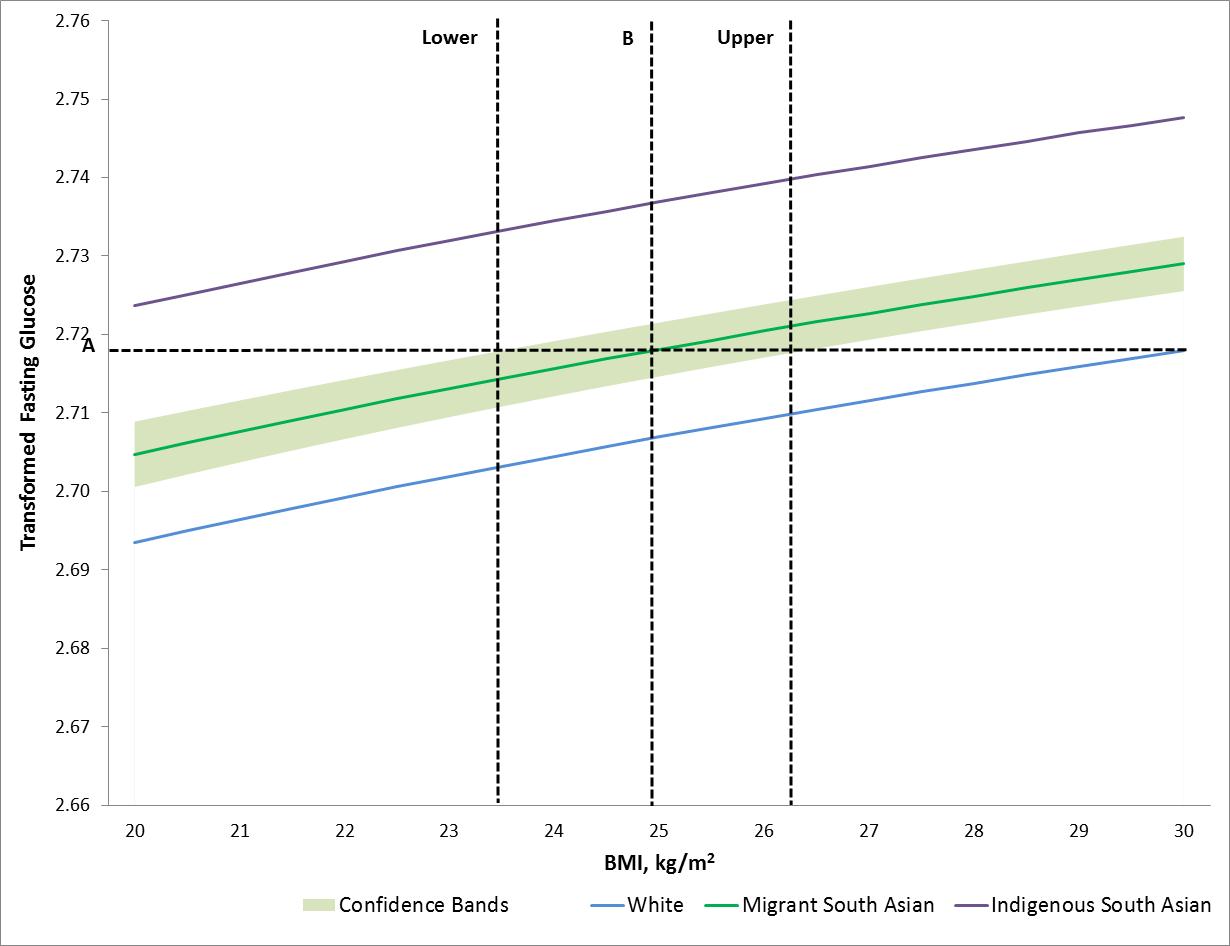
**

**Figure 2.** The age-adjusted relationship between body mass index (BMI) and fasting glucose by ethnic group with confidence bands shown for the migrant South Asian group.
