# Supplementary material for: Body Mass Index and Waist Circumference Cut-Points in Multi-Ethnic Populations from the UK and India: The ADDITION-Leicester, Jaipur Heart Watch and New Delhi Cross-Sectional Studies
Source: PLoS One. 2014 Mar 5;9(3):e90813. doi: 10.1371/journal.pone.0090813 (PMC3944886; doi:10.1371/journal.pone.0090813)

## Table S1. Unadjusted and adjusted models used to find cut-points.

|  | BMI | | |  | Waist Circumference in Men | | |  | Waist Circumference in Women | | |
| --- | --- | --- | --- | --- | --- | --- | --- | --- | --- | --- | --- |
|  | Variable transformation | Coefficient | Standard error |  | Variable transformation | Coefficient | Standard error |  | Variable transformation | Coefficient | Standard error |
| *Fasting glucose* |  |  |  |  |  |  |  |  |  |  |  |
| Constant | None | 2.6738 | 0.0058 |  | None | 2.6555 | 0.0124 |  | None | 2.6907 | 0.0049 |
| Migrant South Asian | None | 0.0114 | 0.0043 |  | None | -0.0144 | 0.0088 |  | None | 0.0015 | 0.0062 |
| Indigenous South Asian | None | 0.0309 | 0.0106 |  | None | -0.0580 | 0.0238 |  | None | 0.0086 | 0.0162 |
| Adiposity^a^ | Ln(BMI/10) | 0.0602 | 0.0051 |  | None | 0.0008 | 0.0001 |  | (Waist/100)^2 | 0.0481 | 0.0045 |
| Interaction | None | -0.00001 | 0.0001 |  | None | 0.0003 | 0.0001 |  | None | 0.0001 | 0.0001 |
| Age | (Age/10)^-2 | -0.5488 | 0.0638 |  | (Age/10)^-2 | -0.3051 | 0.0979 |  | (Age/10)^-2 | -0.6228 | 0.0824 |
|  |  |  |  |  |  |  |  |  |  |  |  |
| *2-hour glucose* |  |  |  |  |  |  |  |  |  |  |  |
| Constant | None | 2.5514 | 0.0115 |  | None | 2.4566 | 0.0266 |  | None | 2.4427 | 0.0202 |
| Migrant South Asian | None | 0.0265 | 0.0229 |  | None | 0.0230 | 0.0575 |  | None | -0.0148 | 0.0373 |
| Adiposity^a^ | None | 0.0052 | 0.0004 |  | None | 0.0024 | 0.0003 |  | None | 0.0020 | 0.0002 |
| Interaction | None | 0.0010 | 0.0008 |  | None | 0.0004 | 0.0006 |  | None | 0.0007 | 0.0004 |
| Age | (Age/10)^3 | 0.0003 | 0.00002 |  | (Age/10)^3 | 0.0002 | 0.00003 |  | None | 0.0024 | 0.0002 |

Note: Fasting glucose and 2-hour glucose were transformed by adding 10 and then taking the natural logarithm.

^a^ Body Mass Index in the models to find Body Mass Index cut-points. Waist circumference in the models used to find waist circumference cut-points.

**Figure S1.** The age-adjusted relationship between body mass index (BMI) and fasting glucose by ethnic group.


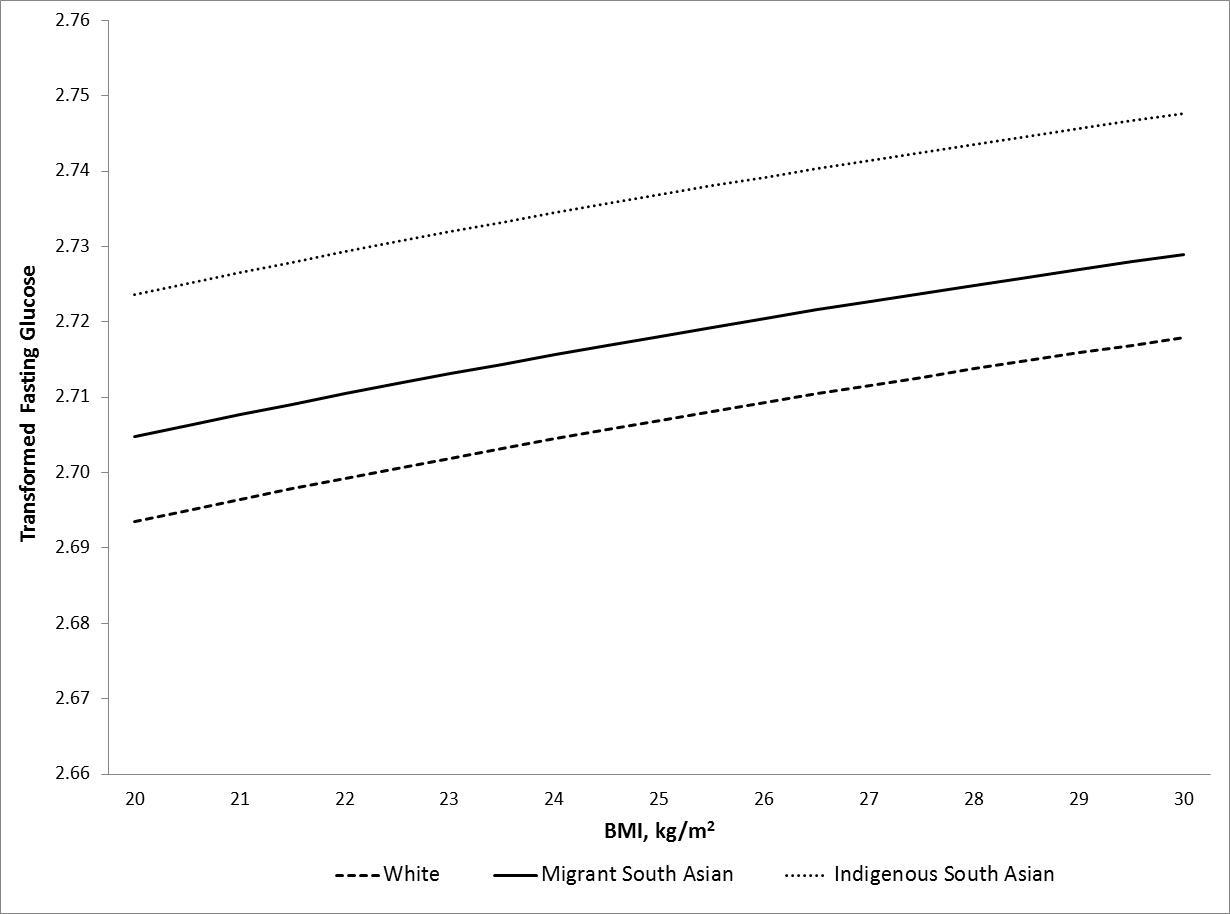


**Figure S2.** The age-adjusted relationship between body mass index (BMI) and 2-hour glucose by ethnic group.

**
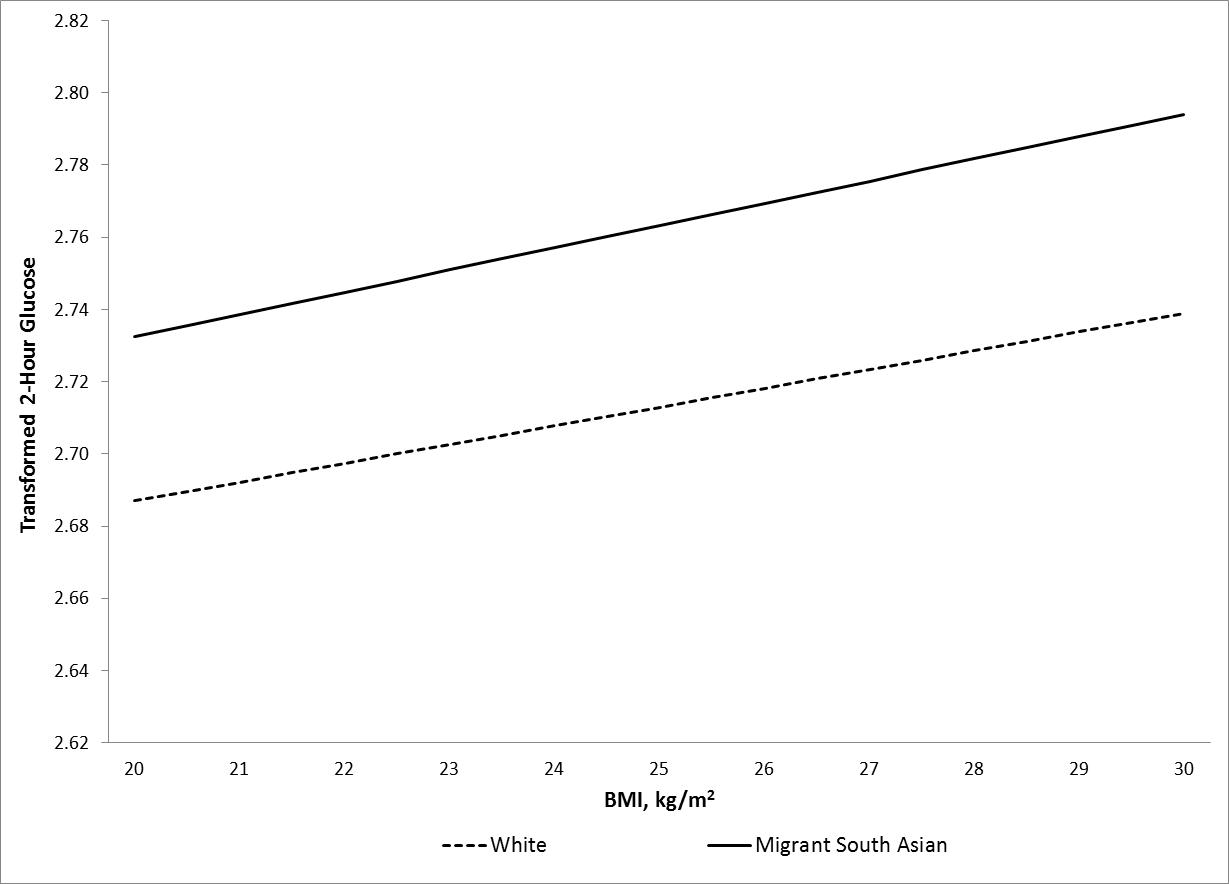
**

**Figure S3.** The age-adjusted relationship between waist circumference and fasting glucose by ethnic group in men.


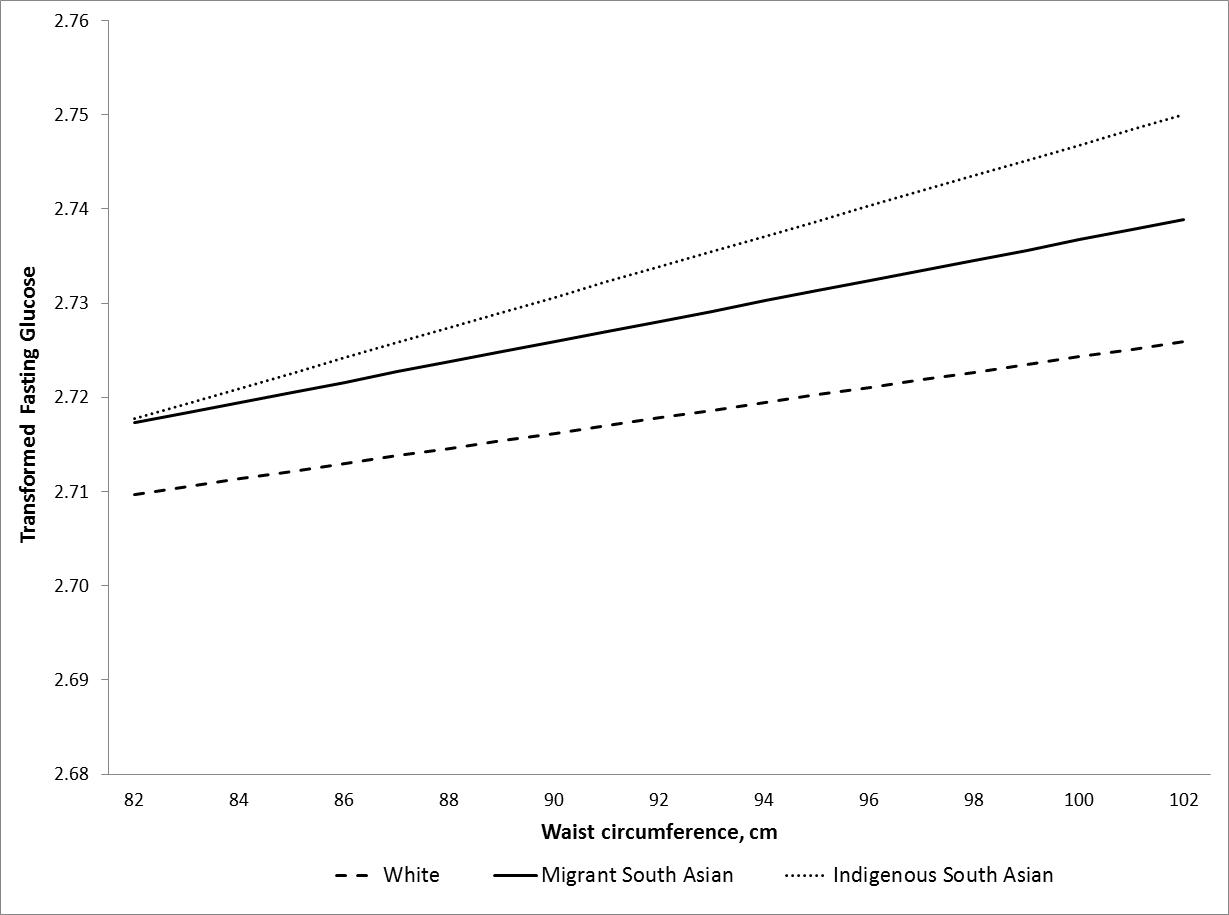


**Figure S4.** The age-adjusted relationship between waist circumference and 2-hour glucose by ethnic group in men.**
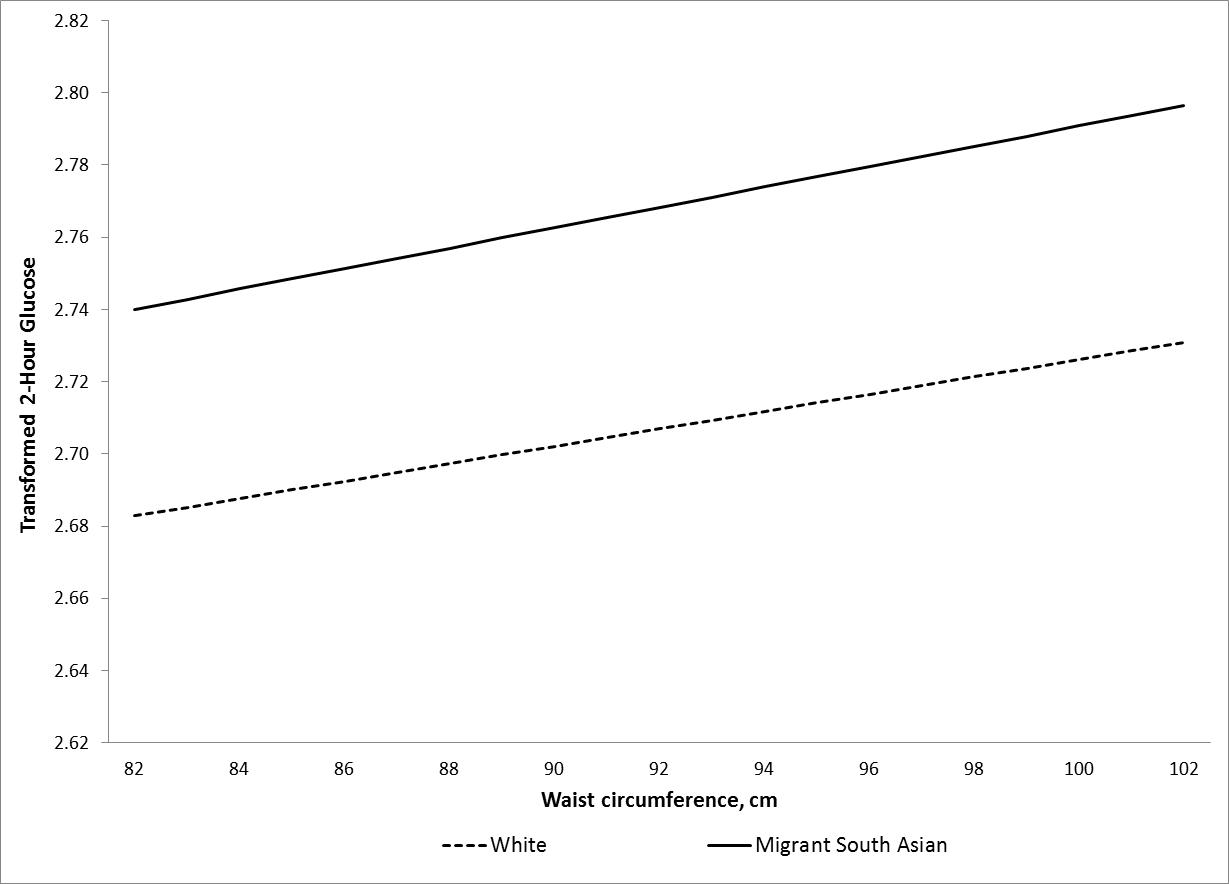
**

**Figure S5.** The age-adjusted relationship between waist circumference and fasting glucose by ethnic group in women.


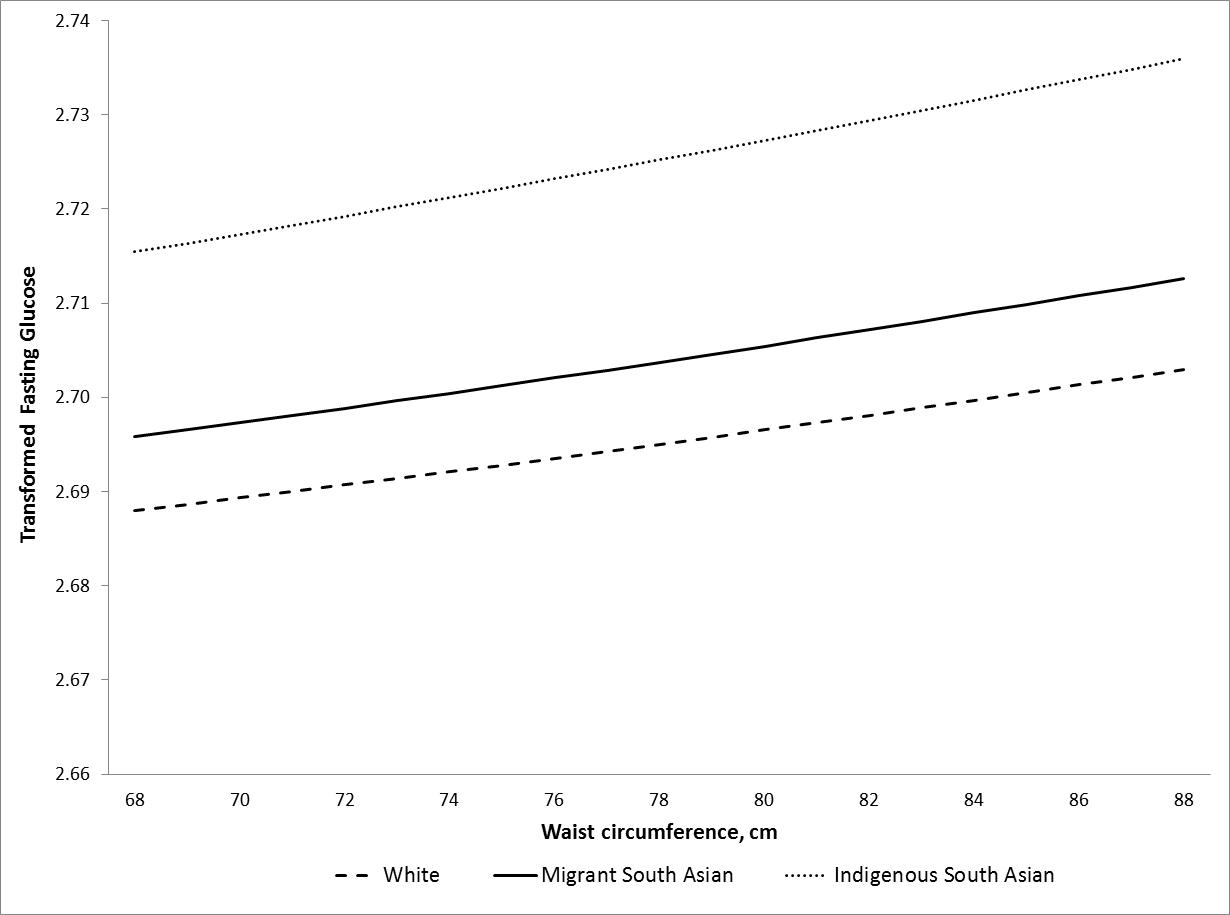


**Figure S6.** The age-adjusted relationship between waist circumference and 2-hour glucose by ethnic group in women.


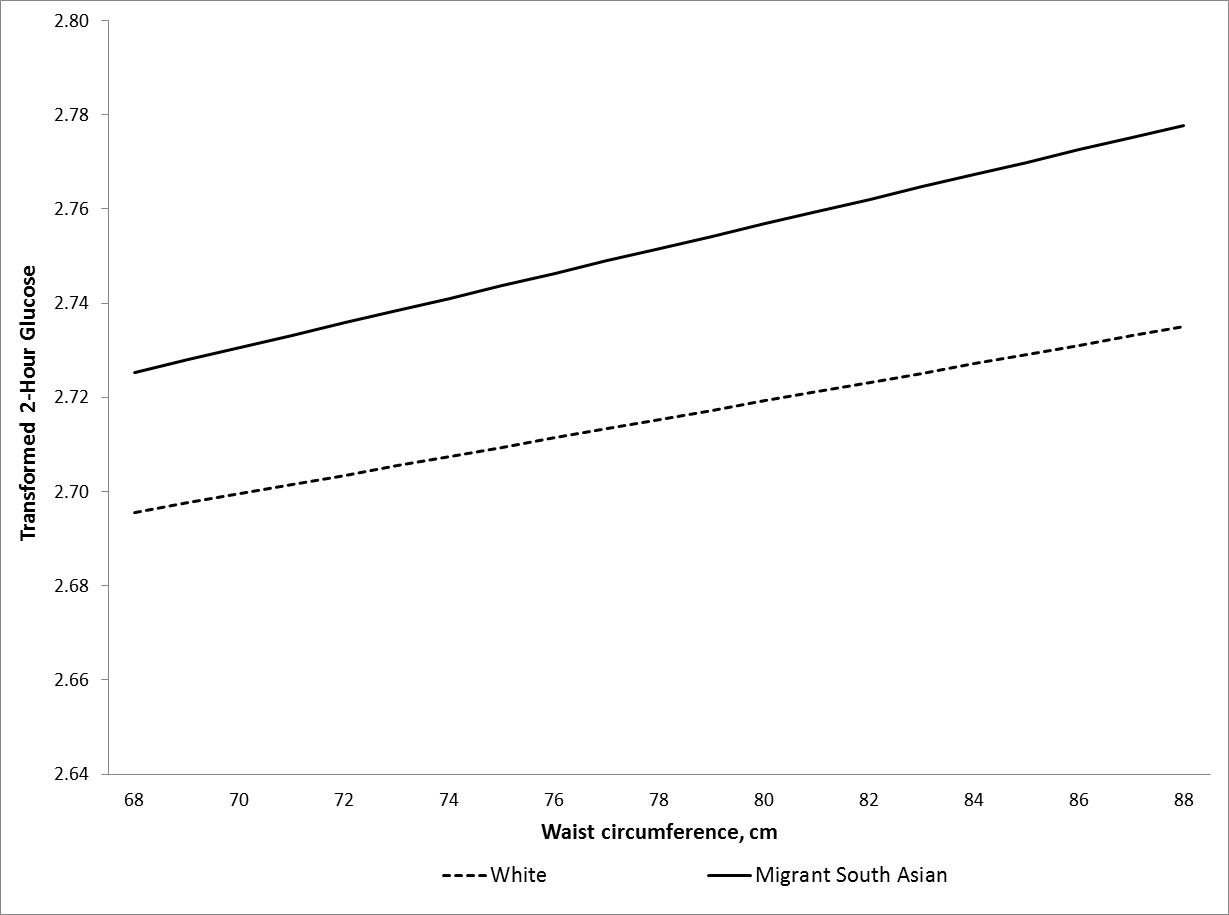

Supplement: File S2 — Table S1 and Figures S1–S6. (DOCX) [file pone.0090813.s002.docx]
